# Dr. Walter D. O. K. Strong

**Published:** 1905-03

**Authors:** 


### OBITUARY.

Dr. Walter D. O. K. Strong, of Fishkill Landing, a graduate of the medical department of Buffalo, 1847, died at his home, January 26, 1905, aged 81 years.

